# Relationship Between COVID‐19 and Retinal Artery Occlusions

**DOI:** 10.1155/joph/5545707

**Published:** 2026-02-22

**Authors:** Tetsuya Muto, Shigeki Machida, Shinichiro Imaizumi, Koju Kamoi

**Affiliations:** ^1^ Department of Ophthalmology, Dokkyo Medical University Saitama Medical Center, Koshigaya, Japan, saitama-med.ac.jp; ^2^ Department of Ophthalmology, Imaizumi Eye Hospital, Koriyama, Japan; ^3^ Department of Ophthalmology, Fukushima Medical University, Fukushima, Japan, fmu.ac.jp; ^4^ Department of Ophthalmology, and Visual Science, Institute of Science Tokyo, Tokyo, Japan, tmd.ac.jp

**Keywords:** branch retinal artery occlusion, central retinal artery occlusion, coronavirus disease 2019

## Abstract

The relationship between coronavirus disease 2019 (COVID‐19) infection or vaccination and retinal artery occlusions (RAOs) remains controversial. COVID‐19 infection or vaccination can sometimes cause thrombin formation. RAOs occur due to thrombin in the retinal artery. The average age of occurrence after COVID‐19 infection was 48.7 ± 17.2 years in central RAO (CRAO) and 41.3 ± 17.8 years in branch RAO (BRAO). After COVID‐19 vaccination, the average age was 54.7 ± 17.1 years in CRAO and 62.3 ± 21.2 years in BRAO. The mean time from COVID‐19 diagnosis to symptom onset was 10.5 ± 9.3 days in CRAO and 48.3 ± 39.6 days in BRAO. After vaccination, the mean time was 7.3 ± 6.8 days in CRAO and 21.0 ± 24.9 days in BRAO. Initial visual acuity (VA) after COVID‐19 infection was 2.53 ± 0.65 in CRAO and 0.09 ± 0.07 in BRAO. Final VA was 2.73 ± 0.12 in CRAO, but data for BRAO were unavailable. After vaccination, initial VA was 2.28 ± 0.97 in CRAO and −0.02 ± 0.16 in BRAO. Final VA was 2.12 ± 1.24 in CRAO and could not be calculated in BRAO. The relationship between COVID‐19 or its vaccination and RAOs was investigated through past case reports. Two types of reports existed regarding RAO incidence after the COVID‐19 pandemic—some indicated an increase, while others found no change. No reports suggested a decrease in RAO occurrence. The current evidence does not clarify the relationship between COVID‐19 or its vaccine and RAOs. However, this relationship cannot be ruled out. Further investigations are necessary, as future infectious disease pandemics may occur.

## 1. Introduction

Coronavirus disease 2019 (COVID‐19) emerged suddenly in China at the end of 2019 and spread worldwide. Severe acute respiratory syndrome coronavirus 2 (SARS‐CoV‐2) is the causative agent of COVID‐19. SARS‐CoV‐2 is a single‐stranded, enveloped RNA virus with a spiral capsid [1]. Initially, it was thought to cause only respiratory dysfunction [2]. However, reports from various countries indicated that COVID‐19 is associated with thrombosis [3, 4]. Thrombosis after COVID‐19 infection has been reported in both veins [5, 6] and arteries [6], with a suggested correlation between retinal vein occlusion (RVO) [7–11] and retinal artery occlusion (RAO) [8–26] in ophthalmology.

COVID‐19 infects various cell types, including pneumonocytes, vascular endothelial cells, and small intestinal epithelial cells, through the angiotensin II‐converting enzyme receptor. Excess complement activity, inflammatory response, disseminated intracapillary coagulation, and cytokine storms exacerbate the coagulation cascade when vascular endothelial cells are directly injured [27]. This condition, known as immune thrombosis, has an unclear mechanism [27].

COVID‐19 vaccination was developed and distributed at an unprecedented speed. However, reports have suggested the possibility of various ocular adverse events [28, 29]. Since these include RVO [29–33] and RAO [29–43], careful attention is required. Although establishing a clear link between ocular adverse events and vaccination is challenging, further analysis is essential.

RAO is an acute condition that typically causes sudden vision loss. If retinal blood flow is not promptly restored, vision loss and visual field defects may become permanent. Although various treatments have been studied [44–46], no standardized treatment has been established, making RAO difficult to manage. Additionally, RAO carries a risk of cardiovascular events [46], such as cerebral stroke. The visual prognosis of central RAO (CRAO) is poor, with decimal visual acuity (VA) usually below 0.05 [47].

In this review, we examine previous reports that were published between 2020 and 2024 focusing on the relationship between COVID‐19 and RAO.

### 1.1. Mechanism of Vascular Occlusion of COVID‐19

Coagulation disorders in COVID‐19 are thought to result from vascular damage caused by viral infection [48]. Various factors, including reduced antithrombogenicity of the vascular endothelium, von Willebrand factor and coagulation factor VIII release, complement activation, increased fibrinogen, and cytokine storms, contribute to thrombosis formation [48]. Thrombi can develop in arteries, veins, and capillaries [48]. Early in the pandemic, 25% of intensive care unit patients with severe COVID‐19 had venous thromboembolism (VTE), which was associated with poor prognosis [49]. These findings were confirmed in clinical studies worldwide and recognized as COVID‐19–associated coagulopathy (CAC) [50]. CAC is not merely the activation of the coagulation and fibrinolysis system but involves multiple pathways, including inflammation, immune response, complement activation, and the kinin–kallikrein system. It is a complex pathophysiology involving leukocytes, platelets, and vascular endothelial cells [48, 50].

Several reports have documented severe thrombosis after COVID‐19 vaccination [51–59]. COVID‐19 vaccines interact with platelets or platelet factor 4 (PF4), leading to vaccine‐induced immune thrombotic thrombocytopenia (VITT). The proposed mechanisms include the formation of autoantibodies against PF4, antibodies induced by free deoxyribonucleic acid (DNA) in the vaccine that cross‐react with PF4, and platelet activation due to adenovirus binding. VITT may explain vaccine‐related vascular occlusions [60].

Since postvaccine thrombosis is often accompanied by thrombocytopenia, it is referred to as thrombosis with thrombocytopenia syndrome (TTS) [53] or VITT [54]. Although rare, adenovirus vector COVID‐19 vaccines have been associated with thrombosis in cerebral and visceral veins [55]. Postvaccine thrombosis has similarities to heparin‐induced thrombocytopenia (HIT) and is often associated with HIT antibody positivity [56]. Additionally, autoimmune coagulation disorders such as idiopathic thrombocytopenic purpura (ITP) [57], thrombotic thrombocytopenic purpura (TTP) [58], and acquired coagulation factor inhibitors [59] have been reported after COVID‐19 vaccination.

### 1.2. CRAO After COVID‐19 Infection

CRAO cases after COVID‐19 infection are shown in Tables 1 and 2 [12, 13, 15–18, 20–23, 25, 26]. The average age was 48.7 ± 17.2 years, with a male‐to‐female ratio of 9:4. CRAO occurred in the right eye in five cases, the left eye in six cases, and both eyes in two cases. According to Lee, among 91 patients with acute nonarteritic CRAO, 62.6% were male, and the average age was 66.4 years [60]. The average age in this study was notably lower due to the inclusion of a 6‐year‐old boy [12]. Lee also reported a higher incidence of CRAO in males after COVID‐19 infection [60]. The mean time from COVID‐19 diagnosis to CRAO symptom onset was 10.5 ± 9.3 days. Coagulation factor levels were elevated in most cases.

**TABLE 1:** Characteristics of patients with CRAO after COVID‐19 infection.

No.	Authors	Age	Sex	Laterality	Time between COVID‐19 diagnosis and symptom onset (days)	Abnormal blood test findings
1	Abbati et al. [12]	6	F	L	0	CRP (1.12 mg/L)
				R	0	
2	Acharya et al. [13]	60	M	R	12	Not listed
3	Bapaye et al. [15]	42	M	B	13	Normal
4	Been et al. [16]	38	M	L	Unknown	Not listed
5	de Oliveira et al. [17]	68	F	L	23	D‐dimer (1,386 μg/L)
						CRP (22.9 mg/L)
						Fibrinogen (587 mg/dL)
6	Heidarzadeh et al.[18]	44	M	L	21	Normal
7	Larochelle et al. [20]	68	M	R	0	Not listed
8	Lekha et al. [21]	47	M	R	Unknown	Not listed
9	Montesel et al. [22]	59	M	L	10	Normal
10	Ucar et al. [23]	54	M	L	Not listed	Fibrinogen (405.1 mg/dL)
						CRP (128.29 mg/L)
						D‐dimer (1,041 μg/L)
						Ferritin (458.53)
						Platelet count (486 × 10^9^)
11	Yalçınbayır et al. [25]	48	F	R	14	Elevated D‐dimer levels
						Elevated fibrinogen levels
						Elevated factor VIII levels
						Elevated von Willebrand factor levels
						Decreased antithrombin levels
		66	M	L	22	Elevated D‐dimer levels
						Elevated fibrinogen levels
						Elevated factor VIII levels
						Elevated von Willebrand factor levels
12	Eldakkak et al. [26]	33	F	R	0	Not listed

*Note:* F, female; L, left; M, male; R, right.

Abbreviations: CRAO, central retinal artery occlusion; CRP, C‐reactive protein.

**TABLE 2:** Visual acuity and treatment of patients with CRAO after COVID‐19 infection.

No.	Authors	BCVA at initial visit	Treatment	Final BCVA
1	Abbati, et al. [12]	HM	Heparin	CF
		NLP	Intravenous steroid	NLP
			Oral prednisolone	
2	Acharya, et al. [13]	NLP	Not listed	Not listed
3	Bapaye, et al. [15]	LP	Not listed	LP
4	Been et al. [16]	CF	Not listed	Not listed
5	de Oliveira, et al. [17]	20/400	Ocular massage	CF
			Hypotensive eye drops	
6	Heidarzadeh, et al. [18]	LP	Oral prednisolone, PRP	NLP
7	Larochelle, et al. [20]	LP	Not listed	Not listed
8	Lekha, et al. [21]	6/36	Not listed	Not listed
9	Montesel, et al. [22]	LP	Not listed	CF
10	Ucar, et al. [23]	CF	20% mannitol	Not listed
			Anterior chamber paracentesis	
			Topical brimonidine	
			Dorzolamide/timolol	
			Moxifloxacin/dexamethasone combination drops	
			Oral acetazolamide	
			Oral aspirin	
11	Yalçınbayır, et al. [25]	HM	Anterior chamber paracentesis	HM
		LP	Amoxicillin clavulanic acid	LP
			Prednisolone	
12	Eldakkak, et al. [26]	NLP	Not listed	HM

Abbreviations: BCVA, best‐corrected visual acuity; CF, counting fingers; CRAO, central retinal artery occlusion; HM, hand motion; LP, light perception; NLP, no light perception; PRP, pan‐retinal photocoagulation.

The initial logarithmic VA was 2.53 ± 0.65, based on previous studies [61]. Treatment was inconsistent, and the final logarithmic VA was 2.73 ± 0.12. Some patients had no light perception (NLP) at the initial visit, and some remained NLP at follow‐up. The visual outcomes were extremely poor. Shah et al. reported that 447 of 484 patients who presented within 30 days had a VA of ≤ 20/200 [62]. Of the 441 patients with documented follow up, 380 (86.2%) maintained the same VA long‐term. A previous study [62] suggested that shorter symptom duration before treatment improved prognosis. However, Shah et al. found that this duration did not influence final VA outcomes [62]. St. Peter et al. reported that hyperbaric oxygen therapy might be effective for nonarteritic CRAO, especially when initiated early [63]. They emphasized the importance of immediate treatment [63]. Currently, no single treatment has proven consistently effective for CRAO, and visual outcomes remain poor.

### 1.3. CRAO After COVID‐19 Vaccination

CRAO cases after COVID‐19 vaccination are shown in Tables 3, 4, 5, and 6 [34–36, 40–43]. The average age was 54.7 ± 17.1 years, with a male‐to‐female ratio of 4:3. Among Lee’s 91 patients with acute nonarteritic CRAO, 62.6% were male, and the average age was 66.4 years [60]. This reported age [60] appears higher than the average age in Tables 3, 4, 5, and 6. The time from COVID‐19 vaccination to CRAO symptom onset ranged from 1 to 21 days, with a mean of 7.3 ± 6.8 days.

**TABLE 3:** Characteristics of patients with CRAO after COVID‐19 vaccination.

No.	Authors	Age	Sex	Laterality	Time between vaccination and symptom onset (days)
1	Abdin, et al. [34]	76	F	L	2
2	Chen, et al. [35]	40	F	L	21
3	Chow, et al. [36]	70	M	R	5
4	Thakar, et al. [40]	44	M	L	10
5	Wang, et al. [41]	70	M	R	5
6	Yamagishi, et al. [42]	33	F	L	1
7	Moses, et al. [43]	50	M	R	7

*Note:* F, female; L, left; M, male; R, right.

Abbreviation: CRAO, central retinal artery occlusion.

**TABLE 4:** COVID‐19 vaccination of patients with CRAO.

No.	Authors	Vaccine type	Number of vaccine dose
1	Abdin, et al. [34]	Adenoviruses	First
2	Chen, et al. [35]	mRNA	First
3	Chow, et al. [36]	mRNA	First
4	Thakar, et al. [40]	Inactivated	Second
5	Wang, et al. [41]	mRNA	Not listed
6	Yamagishi, et al. [42]	mRNA	Second
7	Moses, et al. [43]	Adenoviruses	Not listed

Abbreviation: CRAO, central retinal artery occlusion.

**TABLE 5:** Blood test findings and visual acuity of patients with CRAO after COVID‐19 vaccination.

No.	Authors	Abnormal blood test findings	BCVA at initial visit
1	Abdin, et al. [34]	Normal	HM
2	Chen, et al. [35]	LDL (125 mg/dL)	20/40
3	Chow, et al. [36]	Unremarkable	CF
4	Thakar, et al. [40]	ESR (28 mm/h)	LP
5	Wang, et al. [41]	Anti‐PF 4 (73.34 ng/mL)	CF
6	Yamagishi, et al. [42]	Not listed	Not listed
7	Moses, et al. [43]	ESR (120 mm/h)	HM
		CRP (229 mg/L)	
		Cr (1.4 mg/dL)	

Abbreviations: BCVA, best‐corrected visual acuity; CF, counting fingers; Cr, creatinine; CRAO, central retinal artery occlusion; CRP, C‐reactive protein; ESR, erythrocyte sedimentation rate; HM, hand motion; LDL, low‐density lipoprotein; LP, light perception; PF, platelet factor.

**TABLE 6:** Treatment and final visual acuity of patients with CRAO after COVID‐19 vaccination.

No.	Authors	Treatment	Final BCVA
1	Abdin, et al. [34]	Ocular massage	Not listed
		Pentoxifylline IV (500 mL)	
		Dorzolamide eye drop	
		Aspirin (100 mg)	
2	Chen, et al. [35]	Heparin	NLP
3	Chow, et al. [36]	Clopidogrel and hyperbaric oxygen therapy	CF
4	Thakar, et al. [40]	Not listed	Not listed
5	Wang, et al. [41]	Hyperbaric oxygen therapy	CF
		Topical antiglaucoma drops	
6	Yamagishi, et al. [42]	Intravenous D‐mannitol	1.2
		Acetazolamide	
		10‐min ocular massage	
7	Moses, et al. [43]	Pulse‐dose steroids	CF
		Cyclophosphamide	
		Mepolizumab	

*Note:* F, female; L, left; M, male; R, right; w, week.

Abbreviations: BRAO, branch retinal artery occlusion; CRP, C‐reactive protein; PT, prothrombin time; WBC, white blood cell.

The COVID‐19 vaccines used were manufactured by Pfizer, Moderna, AstraZeneca, and Bharat Biotech, as shown in Table 4.

Although some blood test abnormalities were observed, they were not a characteristic feature of this subset of patients. VA at the initial visit was generally poor, except for one case with a VA of 20/40. The initial logarithmic VA was 2.28 ± 0.97, based on prior data [61]. Some patients had NLP at their final visit, with only one case showing partial visual recovery, reaching a decimal VA of 1.2. Treatment was inconsistent, and the final logarithmic VA was 2.12 ± 1.24.

### 1.4. Branch RAO (BRAO) After COVID‐19 Infection

Published cases of BRAO after COVID‐19 infection are summarized in Tables 7 and 8 [9, 14, 19, 24]. The average patient age was 41.3 ± 17.8 years, and all cases occurred in males. The average time from COVID‐19 diagnosis to BRAO symptom onset was 48.3 ± 39.6 days. No identifiable patterns were observed regarding the normalization of blood test results.

**TABLE 7:** Characteristics of patients with BRAO after COVID‐19 infection.

No.	Authors	Age	Sex	Laterality	Time between COVID‐19 diagnosis and symptom onset (days)	Abnormal blood test findings
1	Panigrahi, et al. [9]	23	F	R	21	PT (16 s)
						D‐dimer (732 ng/mL)
						Serum ferritin (411 μg/L)
2	Ateş, et al. [14]	34	F	R	107	CRP levels slightly abnormal
						Lupus anticoagulant levels slightly abnormal
						Fibrinogen levels slightly abnormal
						D‐dimer levels slightly abnormal
						Ferritin levels slightly abnormal
3	Hirosawa, et al. [19]	43	F	R	30	WBC (11.7 × 10^9^/L)
4	Uzun, et al. [24]	65	F	L	5 weeks	D‐dimer (1.76 mg/L)

*Note:* F, female; L, left; M, male; R, right; w, week.

Abbreviations: BRAO, branch retinal artery occlusion; CRP, C‐reactive protein; PT, prothrombin time; WBC, white blood cell.

**TABLE 8:** Visual acuity and treatment of patients with BRAO after COVID‐19 infection.

No.	Authors	BCVA at initial visit	Treatment	Final BCVA
1	Panigrahi et al. [9]	6/9	Systemic anticoagulants	Not listed
2	Ateş et al. [14]	10/10	Hyperbaric oxygen therapy	Not listed
3	Hirosawa et al. [19]	20/25	Alprostadil	Not listed
4	Uzun et al. [24]	20/25	Aspirin	Not listed

Abbreviations: BCVA, best‐corrected visual acuity; BRAO, branch retinal artery occlusion.

At the initial visit, the average VA was 0.092 ± 0.071, which was significantly better than that in CRAO cases following COVID‐19 infection (*p* < 0.001) (Table 8). Due to the lack of an established treatment, management strategies varied. Final VA outcomes were not reported for all cases, limiting a comprehensive assessment of treatment efficacy (Table 8).

### 1.5. BRAO After COVID‐19 Vaccination

Reported cases of BRAO after COVID‐19 vaccination are summarized in Tables 9, 10, 11, and 12 [32, 37, 39]. The average patient age was 62.3 ± 21.2 years, and the mean time from vaccination to BRAO symptom onset was 21.0 ± 24.9 days. The average age of patients with CRAO after COVID‐19 vaccination was 55.5 ± 18.5 years, with no significant difference from BRAO cases postvaccination (*p* = 0.49). Similarly, the time from vaccination to symptom onset in BRAO cases (21.0 ± 24.9 days) was not significantly different from CRAO cases (*p* = 0.24).

**TABLE 9:** Characteristics of patients with BRAO after COVID‐19 vaccination.

No.	Authors	Age	Sex	Laterality	Time between vaccination and symptom onset (days)
1	Girbardt et al. [32]	38	M	R	3
2	Ishibashi et al. [37]	38	F	R	15
		80	M	R	42
		86	M	L	4
		57	F	R	61
3	Murata et al. [39]	75	M	R	1

*Note:* F, female; L, left; M, male; R, right.

Abbreviation: BRAO, branch retinal artery occlusion.

**TABLE 10:** Vaccination of patients with BRAO after COVID‐19 vaccination.

No.	Authors	Vaccine type	Number of vaccine doses
1	Girbardt, et al. [32]	mRNA	Second
2	Ishibashi, et al. [37]	mRNA	First
		mRNA	Second
		mRNA	Second
		mRNA	Second
3	Murata, et al. [39]	mRNA	Fourth

Abbreviation: BRAO, branch retinal artery occlusion.

**TABLE 11:** Blood test findings and visual acuity of patients with BRAO after COVID‐19 vaccination.

No.	Authors	Abnormal blood test findings	BCVA at initial visit
1	Girbardt, et al. [32]	Normal	Not listed
2	Ishibashi, et al. [37]	Not listed	20/13
		Not listed	20/20
		Not listed	20/25
		Not listed	20/13
3	Murata, et al. [39]]	Normal except those affected by diabetes	0.7

Abbreviations: BCVA, best‐corrected visual acuity; BRAO, branch retinal artery occlusion.

**TABLE 12:** Treatment and final visual acuity of patients with BRAO after COVID‐19 vaccination.

No.	Authors	Treatment	Final BCVA
1	Girbardt, et al. [32]	Aspirin	Not listed
		Simvastatin	
2	Ishibashi, et al. [37]	Not listed	Not listed
		Not listed	Not listed
		Not listed	Not listed
		Not listed	Not listed
3	Murata, et al. [39]	No treatment	0.7

Abbreviations: BCVA, best‐corrected visual acuity; BRAO, branch retinal artery occlusion.

All BRAO cases following COVID‐19 vaccination involved the Pfizer–BioNTech vaccine (Table 10).

The average initial VA was −0.025 ± 0.16, which was significantly better than that of CRAO cases after COVID‐19 vaccination (*p* < 0.001) (Table 11). However, no significant difference was observed between BRAO cases after COVID‐19 infection and BRAO cases after vaccination (*p* = 0.19).

Due to the lack of reported treatment strategies and final VA outcomes in most cases, a comprehensive evaluation was not possible (Table 12).

### 1.6. From Big Data Studies

Table 13 summarizes big data studies that reported the association between RAO or RVO and COVID‐19 or COVID‐19 vaccination [64–71]. Au et al. analyzed 15 patients with CRAO and found that none of them contracted SARS‐CoV‐2 infection before their CRAO disease episodes [64]. Although Yeung et al. reported 15 patients with RAO after vaccination, they did not mention the correlation between RAO and COVID‐19 vaccination [65]. Although Singh et al. concluded that most cases of RAO (48.27%) were reported within the first week after vaccination, they did not mention the correlation between RAO and COVID‐19 vaccination [66]. Li et al. examined 7,318,437 cases and found that the risk of RAO and RVO significantly increased during the first 2 weeks after vaccination and persisted for 12 weeks [67]. Additionally, individuals who received first and second doses of BNT162b2 and mRNA‐1273 had significantly increased risk of RVO 2 years after vaccination [67]. Al‐Moujahed et al. investigated 442,186 patients and demonstrated that the risk of RAO increased during the first few months of the COVID‐19 pandemic [68]. Feltgen et al. examined 421 patients with RAO and RVO and found no evidence of any association between SARS‐CoV‐2 vaccination and a higher RAO and RVO risk [69]. Park et al. reported RAO incidence rates of 11.7 and 12.0 per 100,000 people per year for 2018‐2019 and 2020‐2021, respectively [70]. They concluded that SARS‐CoV‐2 infection did not significantly increase the incidence of RAO [70]. Park et al. examined 8,418,590 patients and observed no increase in the hazard ratio of RAO relative to COVID‐19 or COVID‐19 vaccination except for a possible increase in the hazard ratio of RAO in women vaccinated with mRNA‐1273 [71]. As these reports [64–71] are regarding big data, the results are reliable. However, the correlation between RAO and COVID‐19 or COVID‐19 vaccination is still unclear.

**TABLE 13:** Correlation between RAO and COVID‐19 infection or vaccination from big data studies.

No.	Authors	Number of patients	Conclusions
1	Au et al. [64]	15 patients with CRAO	COVID‐19–related CRAO cases were just coincident.
2	Yeung et al. [65]	15 patients with RAO	Correlation between RAO and COVID‐19 vaccination was not mentioned.
3	Singh et al. [66]	433 patients with RAO	Correlation between RAO and COVID‐19 vaccination was not mentioned.
4	Li et al. [67]	1,478,132	The risk of RAO and retinal vein occlusion significantly increased during the first 2 weeks after vaccination.
5	Al‐Moujahed et al. [68]	442,186	The number of patients with RAO increased during the first few months of the COVID‐19 pandemic.
6	Feltgen et al. [69]	421 patients with RAO and retinal vein occlusion	No evidence of any association was found between SARS‐CoV‐2 vaccination and a higher risk for RAO and retinal vein occlusion.
7	Park et al. [70]	21,696 patients with RAO	SARS‐CoV‐2 infection did not significantly increase the incidence of RAO.
8	Park et al. [71]	8,418,590 patients	No increase in the hazard ratio of RAO relative to COVID‐19 or COVID‐19 vaccination, except for a possible increase in the hazard ratio of RAO among women vaccinated with mRNA‐1273.

### 1.7. COVID‐19 Pandemic and RAOs

We will now discuss the mechanisms involved in RAO pathogenesis. RAO is primarily caused by atherosclerosis, which leads to plasma component leakage from blood vessels, resulting in retinal hemorrhage and edema. If the lesion remains outside the arcade area, symptoms may be absent. However, once it extends into this region, VA deteriorates rapidly. Risk factors for RAO include older age, hypertension, diabetes, hyperlipidemia, smoking, syphilis, and glaucoma [72]. Additionally, RAO can be secondary to underlying conditions such as atrial fibrillation, valvular heart disease, internal carotid artery stenosis, and hypertension‐induced arteriosclerosis [47]. In younger individuals, RAO may be associated with antiphospholipid syndrome or, in rare cases, oral contraceptive use. Diagnosis requires medical imaging and systemic management by internal medicine specialists.

Thrombosis can occur in various arterial locations, with the highest incidence in the extremities (39%), followed by cerebral vessels (24%), large vessels (19%), coronary arteries (9%), and the superior mesenteric artery (8%), with the remaining 1% involving other vessels [73]. Malas et al. reported an overall incidence of COVID‐19–associated VTE of 21% [74]. VTE incidence was 5% among general ward patients but increased to 31% in ICU patients [74]. When thrombosis affects the retinal artery, it manifests as RAO.

Beyond RAO, the pandemic has been associated with an increased incidence of Vogt–Koyanagi–Harada disease. Before the pandemic, 73 cases were reported over 60 months, compared to 53 cases over 33 months postpandemic [75]. Liang et al. observed a decline in overall eye injuries and notable changes in the pattern of ocular trauma during the pandemic [76]. Increased screen time during lockdowns may have contributed to a rise in dry eye–related complaints among students [77]. Mask‐associated dry eye also became more prevalent [78]. The incidence of endophthalmitis following intravitreal injections remained stable [79], but postvitrectomy endophthalmitis, particularly cases linked to oral bacteria, increased [80]. Shi et al. reported a rise in optic neuritis cases during the pandemic [81], along with an increase in acute primary angle closure, though the underlying cause remains unclear [82].

## 2. Conclusion

This systematic review summarizes reported cases of RAO secondary to COVID‐19 infection or vaccination. However, no factors definitively establish an association between RAO and COVID‐19 infection or vaccination. The possibility of a link cannot be denied, but no reports have shown a decrease in RAO cases after the pandemic. Many diseases were affected by the COVID‐19 pandemic. Therefore, further epidemiological studies with more data are needed.

## Funding

The authors declare that they have received no funding.

## Conflicts of Interest

The authors declare no conflicts of interest.

## Data Availability

The datasets used and/or analyzed during this study are available from the corresponding author upon reasonable request.
